# Automated imaging response evaluation system: Prototype software tool for standardized radiographic response assessment in glioblastoma clinical trials

**DOI:** 10.1093/nop/npag039

**Published:** 2026-05-14

**Authors:** Ashley Teraishi, Francesco Sanvito, Catalina Raymond, Nicholas S Cho, Jason Norris, Sani Gandhi, Alexander R Cohen, Yashaar Hafizka, Audrey Luo, Hao-Wen Sim, Lucas P Wachsmuth, Sonia Yip, Eng-Siew Koh, Merryn Hall, David M Ashley, Mark A Rosenthal, Elizabeth J Hovey, Hui K Gan, Margaret O Johnson, Elizabeth H Barnes, John R Simes, Zarnie Lwin, Mustafa Khasraw, Noriko Salamon, Timothy F Cloughesy, Benjamin M Ellingson

**Affiliations:** UCLA Brain Tumor Imaging Laboratory (BTIL), Center for Computer Vision and Imaging Biomarkers, University of California, Los Angeles, Los Angeles, CA, USA; Department of Radiological Sciences, David Geffen School of Medicine, University of California, Los Angeles, Los Angeles, CA, USA; UCLA Brain Tumor Imaging Laboratory (BTIL), Center for Computer Vision and Imaging Biomarkers, University of California, Los Angeles, Los Angeles, CA, USA; Department of Radiological Sciences, David Geffen School of Medicine, University of California, Los Angeles, Los Angeles, CA, USA; UCLA Brain Tumor Imaging Laboratory (BTIL), Center for Computer Vision and Imaging Biomarkers, University of California, Los Angeles, Los Angeles, CA, USA; Department of Radiological Sciences, David Geffen School of Medicine, University of California, Los Angeles, Los Angeles, CA, USA; UCLA Brain Tumor Imaging Laboratory (BTIL), Center for Computer Vision and Imaging Biomarkers, University of California, Los Angeles, Los Angeles, CA, USA; Department of Radiological Sciences, David Geffen School of Medicine, University of California, Los Angeles, Los Angeles, CA, USA; Department of Bioengineering, Henry Samueli School of Engineering and Applied Science, University of California, Los Angeles, Los Angeles, CA, USA; Medical Scientist Training Program, David Geffen School of Medicine, University of California, Los Angeles, Los Angeles, CA, USA; Department of Radiological Sciences, David Geffen School of Medicine, University of California, Los Angeles, Los Angeles, CA, USA; Department of Neurology, David Geffen School of Medicine, University of California, Los Angeles, Los Angeles, CA, USA; Department of Neurology, David Geffen School of Medicine, University of California, Los Angeles, Los Angeles, CA, USA; UCLA Brain Tumor Imaging Laboratory (BTIL), Center for Computer Vision and Imaging Biomarkers, University of California, Los Angeles, Los Angeles, CA, USA; Department of Radiological Sciences, David Geffen School of Medicine, University of California, Los Angeles, Los Angeles, CA, USA; UCLA Brain Tumor Imaging Laboratory (BTIL), Center for Computer Vision and Imaging Biomarkers, University of California, Los Angeles, Los Angeles, CA, USA; Department of Radiological Sciences, David Geffen School of Medicine, University of California, Los Angeles, Los Angeles, CA, USA; NHMRC Clinical Trials Centre, University of Sydney, Sydney, Australia; South Western Sydney Clinical School, Faculty of Medicine, University of New South Wales, Sydney, Australia; Department of Medical Oncology, The Kinghorn Cancer Centre, Sydney, Australia; Department of Medical Oncology, Chris O’Brien Lifehouse, Sydney, Australia; The Brain Tumor Immunotherapy Program, Duke University School of Medicine, Duke University Medical Center, Durham, NC, USA; NHMRC Clinical Trials Centre, University of Sydney, Sydney, Australia; South Western Sydney Clinical School, Faculty of Medicine, University of New South Wales, Sydney, Australia; Department of Radiation Oncology, Liverpool Hospital, Sydney, Australia; NHMRC Clinical Trials Centre, University of Sydney, Sydney, Australia; The Preston Robert Tisch Brain Tumor Center, Duke University School of Medicine, Duke University Medical Center, Durham, NC, USA; Department of Medical Oncology, Peter MacCallum Cancer Centre, Melbourne, Australia; South Western Sydney Clinical School, Faculty of Medicine, University of New South Wales, Sydney, Australia; Department of Medical Oncology, Prince of Wales Hospital, Sydney, Australia; Department of Medical Oncology, Austin Hospital, Melbourne, Australia; The Preston Robert Tisch Brain Tumor Center, Duke University School of Medicine, Duke University Medical Center, Durham, NC, USA; NHMRC Clinical Trials Centre, University of Sydney, Sydney, Australia; NHMRC Clinical Trials Centre, University of Sydney, Sydney, Australia; Department of Medical Oncology, Chris O’Brien Lifehouse, Sydney, Australia; Department of Medical Oncology, Royal Brisbane and Women’s Hospital, Brisbane, Australia; NHMRC Clinical Trials Centre, University of Sydney, Sydney, Australia; The Brain Tumor Immunotherapy Program, Duke University School of Medicine, Duke University Medical Center, Durham, NC, USA; The Preston Robert Tisch Brain Tumor Center, Duke University School of Medicine, Duke University Medical Center, Durham, NC, USA; Department of Radiological Sciences, David Geffen School of Medicine, University of California, Los Angeles, Los Angeles, CA, USA; Department of Neurology, David Geffen School of Medicine, University of California, Los Angeles, Los Angeles, CA, USA; UCLA Neuro-Oncology Program, David Geffen School of Medicine, University of California, Los Angeles, Los Angeles, CA, USA; UCLA Brain Tumor Imaging Laboratory (BTIL), Center for Computer Vision and Imaging Biomarkers, University of California, Los Angeles, Los Angeles, CA, USA; Department of Radiological Sciences, David Geffen School of Medicine, University of California, Los Angeles, Los Angeles, CA, USA; Department of Bioengineering, Henry Samueli School of Engineering and Applied Science, University of California, Los Angeles, Los Angeles, CA, USA; Department of Neurosurgery, David Geffen School of Medicine, University of California, Los Angeles, Los Angeles, CA, USA; Department of Psychiatry and Biobehavioral Sciences, David Geffen School of Medicine, University of California, Los Angeles, Los Angeles, CA, USA

## Abstract

The clinical management of patients with brain tumors, as well as the results of clinical trials testing new medications, require the evaluation of tumor changes on MR imaging. After tumor measurements are obtained, the AIRES software application can automatically apply the standardized RANO rules, to ultimately define when the tumor can be considered in a progression phase, as opposed to a stable phase, or in treatment response. This automated tool enables fast, reliable, and reproducible assessments, and therefore has the potential to reduce errors, streamline workflows, and lower costs by minimizing the resources required for training personnel and conducting reads. The standardized Response Assessment in Neuro-Oncology (RANO), modified RANO (mRANO), and RANO 2.0 criteria are utilized for imaging endpoints in glioblastoma clinical trials. After measuring tumor size at each timepoint, the crucial step of categorizing progression, stable disease, and response based on numerical thresholds is currently done manually. “Manual” RANO evaluations are time-consuming, error prone, and require dedicated training. Here, we present the features, installation, and usage of Automated Imaging Response Evaluation System (AIRES), a software application prototype that applies mRANO criteria using externally measured tumor sizes, clinical status, and corticosteroid dose. AIRES labels each timepoint with RANO categories and calculates progression-free survival and objective response rate. As a real-world application on clinical trial datasets, mRANO reads were performed on measurements from 367 brain MRI scans across 41 patients from NUTMEG (NCT04195139), a phase II multicenter clinical trial. Four readers with varying levels of experience achieved a percentage of fully correct RANO reads ranging from 49% to 76%, while AIRES reached 100%, compared to a fifth expert reader. AIRES-based reads were significantly faster than “manual” reads by the expert (37.1 ± 8.6 vs 107.7 ± 58.6 s, *P *< .0001). AIRES is a reliable and time-efficient tool for mRANO assessments and may be adapted for RANO 2.0.

Key Points

- AIRES automatically assigns RANO categories based on tumor sizes measured externally.
- AIRES may reduce the need for personnel training and the risk of manual errors.
- On clinical trial datasets, AIRES-assisted reads were significantly faster than manual reads.

Glioblastoma accounts for approximately 51.5% of all newly diagnosed malignant primary central nervous system tumors^1^ and carries a poor prognosis, with a median survival reportedly around 16-17 months in clinical trial cohorts.^2^^,^^3^ Given the unsatisfactory therapies currently available, new clinical trials test experimental treatment to improve the disease course of these patients. While overall survival (OS) is the gold standard endpoint for glioblastoma clinical trials, progression-free survival (PFS) and objective response rate (ORR) (reflecting the duration of disease control and the response to the treatment, respectively) serve as valuable surrogate endpoints. PFS^4^ and ORR^5^ can be assessed earlier than OS, thus potentially shortening trial durations and increasing the statistical power even when only a subset of patients reach OS within the trial duration. Additionally, PFS and ORR give us additional insights into the disease course beyond OS, since patient survival can be influenced by confounding factors other than tumor growth, including complications and comorbidities.

The Response Assessment in Neuro-Oncology (RANO) criteria, including modified-RANO (mRANO)^6^ and RANO 2.0^7^^,^^8^ are a rigorous and reproducible set of guidelines designed to standardize imaging endpoints in glioblastoma clinical trials. In RANO, the estimation of PFS and ORR primarily rely on MRI-based two-dimensional (bidimensional) or three-dimensional (volumetric) measurements of tumor size changes over time, while also incorporating the evaluation of important clinical variables, such as clinical/neurological status and corticosteroid dose changes, to achieve a comprehensive treatment response assessment.

During clinical trials, local RANO reads are performed by onsite personnel. After the radiographic measurements are obtained, the application of the RANO criteria to derive PFS and ORR from the measurements is rather complex, especially in cases with many timepoints and when confirmation scans are required, and require specialized training to ensure consistent and accurate reads. Additionally, even when performed by trained personnel, RANO reads performed “manually” are very time consuming and inherently error-prone. Although the adjudicated PFS and ORR of a study are typically calculated in the independent radiologic facility (IRF) after imaging data centralization,^9^ prompt and accurate local RANO reads performed in real time are still crucial because the identification of disease progression (PD) warrants the treatment discontinuation and trial exit.

Given the challenges of “manual” RANO reads, AIRES adds value by automating the RANO classification process based on timepoint-specific tumor measurements and clinical variables. AIRES processes tumor measurements that are either calculated previously or performed in real time with the AIRES interface by the operator, and then automatically generates RANO classifications (eg, complete response, partial response, progressive disease, stable disease). AIRES was designed using the mRANO criteria and currently focuses on enhancing tumor measurements. This tool improves the reliability of RANO reads (especially local reads), limiting the risk of human error, reducing the need for specific training, and making the assessment more time-efficient and reproducible. Use cases for RANO reads include clinical trials, retrospective research, and clinical practice.^10^

## Software Overview

### Design and Implementation

AIRES was developed as a standalone application that acts as a “calculator” for radiographic response assessment. The goal of the application is to streamline and speed up RANO read workflows, minimize errors, and facilitate auditing. The application was designed to be flexible with input types, allowing users to manually perform tumor measurements in real time, or to import previously calculated measurements from external tools, such as automated segmentation tools, to ultimately calculate time point responses while factoring in clinical status and steroid usage.

AIRES is a lightweight desktop application built in Python (https://www.python.org/) using the PyQt6 framework (https://www.riverbankcomputing.com/software/pyqt/) for the graphical user interface (GUI) and an SQLite (https://sqlite.org/) database for local, persistent data storage. All data are stored locally on the user’s computer and remain available until the user chooses to delete specific timepoints or clear the database. All required software for running AIRES is included in the installation package, so no additional software (eg, Python, PyQt6, or SQLite) needs to be installed separately. The application optionally integrates with 3D Slicer (https://www.slicer.org),^11^ an open-access and free software platform, to enable real time volumetric segmentations and/or bidimensional measurements, in case the operator prefers to perform measurements on the application rather than with separate dedicated tools (see the paragraph “Options for Tumor Measurements”). Importantly, if the real time measurement option is preferred, 3D Slicer needs to be installed separately by the user, and installation can be prompted directly via the dedicated option on the AIRES menu bar. If 3D Slicer was already installed separately from AIRES, the existing 3D Slicer installation can be linked to AIRES by following the instructions in the software documentation. AIRES has been tested for compatibility with 3D Slicer version 5.8.1.

AIRES is structured based on the Model-View-Controller (MVC) design pattern, which ensures separation between data handling, business logic, and the user interface. This architecture supports code maintainability, readability, and scalability as new features are added. The application is cross-platform compatible and has been tested on macOS (version 13 Ventura and higher) and Windows 11. The software is fully self-contained, using PyInstaller (https://pyinstaller.org), so that no dependencies are required for installation and users can launch the application with a single executable.

### Options for Tumor Measurements

It is important to note that AIRES does not autonomously measure the tumor size, but rather requires the bidimensional or volumetric measurements to be obtained either with separate software products (manually or automatically) or in real-time through the 3D Slicer window integrated with AIRES.

#### External manual measurements

Bidimensional diameters can be manually measured with external imaging tools by a reader, and then entered in AIRES with the user interface or through tabulated files (see the following dedicated paragraphs).

#### External tumor segmentations

Lesion volumes can be derived from tumor segmentations obtained with external software, including manual segmentations or automated AI-based segmentation, for instance with the NS-HGlio deep learning algorithm (Neosoma Inc, Groton, MA, https://neosomainc.com)^12^ or HD-GLIO (https://github.com/CCI-Bonn/HD-GLIO).^13^ Similarly to external manual measurements, the volumes can be entered in AIRES with the user interface or through tabulated files.

#### Real-time measurements with 3D slicer

**Figure 1.**
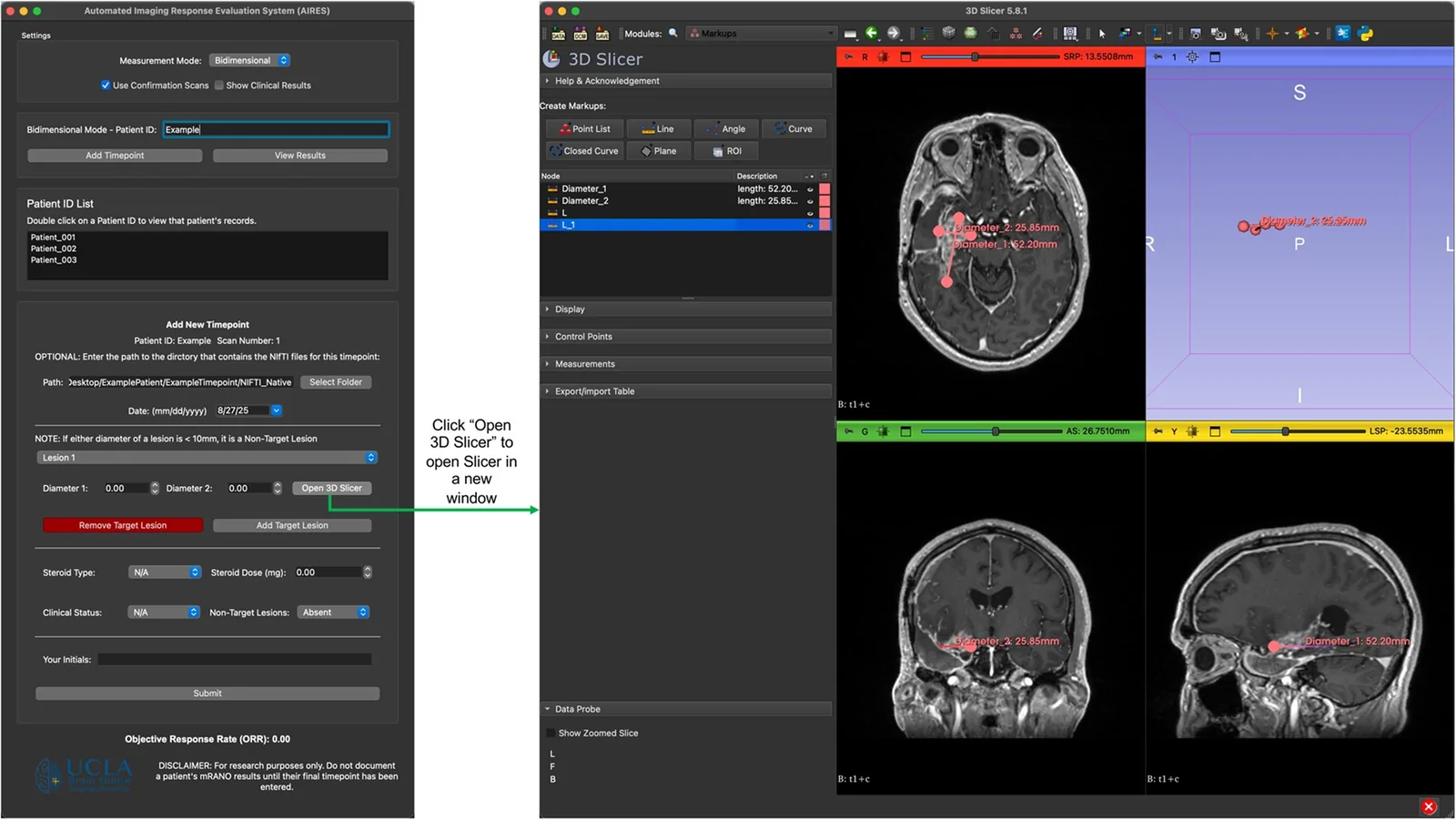
Timepoint input form with 3D slicer integration. Leveraging 3D Slicer deployed within AIRES, users can efficiently perform in-application lesion measurement.

3D Slicer can be launched directly from the AIRES user interface (specifically from the timepoint input form window) to perform new manual measurements or manually refine pre-existing ones in real time (Figure 1). This modality allows for either bidimensional diameters or volumetric segmentations. Once measurements have been made and the 3D Slicer window is closed, the corresponding values are automatically populated into the AIRES timepoint input form.

### Data Entry Through User Interface

**Figure 2.**
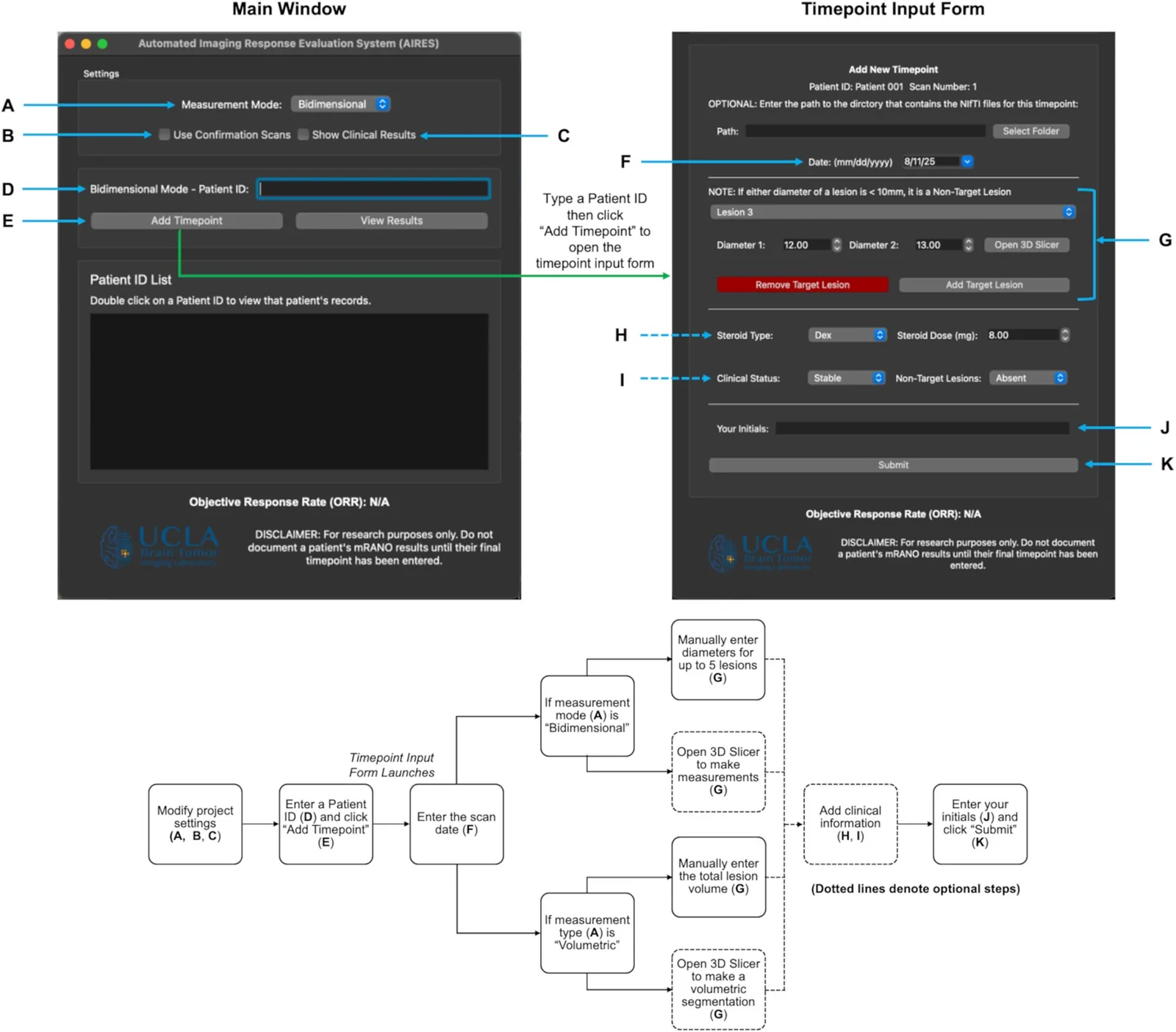
Timepoint input form. Screenshots of the AIRES interface and a flowchart illustrating the process of opening and completing the timepoint input form: a dedicated section for entering comprehensive lesion measurements and associated clinical details.

The application supports multiple methods for data entry, allowing users to tailor the workflow to their specific needs. The most straightforward approach is manually entering individual timepoints through the user interface. Within the application, there is a dropdown menu labeled “Measurement Mode” which allows users to select either “Bidimensional” or “Volumetric” measurements (Figure 2A). Additionally, there is a checkbox that provides the option to perform RANO calculations with or without the requirement of confirmation scans for progression (Figure 2B) and a checkbox to toggle the display of RANO classifications either with integrated clinical data or based on radiographic findings only (Figure 2C). Timepoint-specific information can be added using a dedicated input form. To open the form, users will enter a patient ID (Figure 2D) and click the “Add Timepoint” button (Figure 2E). This will launch the timepoint input form which contains fields for the scan date (Figure 2F) and lesion measurements (Figure 2G). In bidimensional mode, users may enter diameters in millimeters for up to 5 lesions. In volumetric mode, users may enter the total lesion volume in cubic millimeters for up to 5 lesions. Optional fields are also available for steroid type (labeled as “dex”) and steroid dose entered as the dexamethasone equivalent in mg/day (Figure 2H), presence of non-target lesion(s), and clinical/neurological status changes (labeled as “baseline,” “stable,” or “declined”), typically based on the combined assessment of performance status (eg, Karnofsky Performance Scale (KPS)) and neurological tests (eg, the Neurologic Assessment in Neuro-Oncology (NANO)^14^) (Figure 2I). To finalize submission of a timepoint, users will enter their initials (Figure 2J) and click “Submit” (Figure 2K).

It is important to note that AIRES does not provide interpretation of clinical information. Determination of whether a patient’s clinical status is “stable” or “declined” is left to the discretion of the treating physician, and AIRES does not infer clinical status from KPS or NANO scores. Regarding corticosteroid dosing, AIRES internally classifies dose changes of ≤2 mg from baseline as “stable,” with larger changes inferred as “increased” or “decreased.” AIRES does not provide additional interpretation of steroid relevance beyond this categorical classification.

#### Safety checks for data entry

During data entry, AIRES includes validation checks to help prevent common errors. For example, if any entered diameter is less than 10 mm, AIRES alerts the user that it must be entered as a “non-target” lesion (Figure 2I). In addition, when adding new timepoints, if the entered patient ID and date match an existing record, the user is prompted to edit the existing timepoint, which prevents the creation of a duplicate entry.

### Data Entry Through Tabulated Files for Batch Processing

For larger datasets, AIRES supports bulk data entry via comma-separated value (CSV) files using a downloadable template file. This feature is intended to streamline workflows when data has already been compiled (eg, in spreadsheets or other databases) by eliminating the need for repetitive manual entry. Importing a pre-formatted CSV file directly into AIRES reduces the risk of transcription errors that can occur when manually re-typing data. CSV files must follow a predefined structure, with each row representing a single patient timepoint and each column corresponding to a data field. Any data fields with absent data may be filled with “N/A”, additionally for any patients without measurable disease, lesion measurement columns may be filled with “N/A.” A CSV template can be opened from AIRES to ensure correct formatting. The same structure is used for CSV files exported from AIRES, allowing results to be easily shared with collaborators who can quickly import the file into their own AIRES installation to review, verify, and analyze the data. A sample table with mock data is provided in Supplementary Table S1, illustrating the required columns and accepted value formats.

### Timepoint Categorization

**Figure 3.**
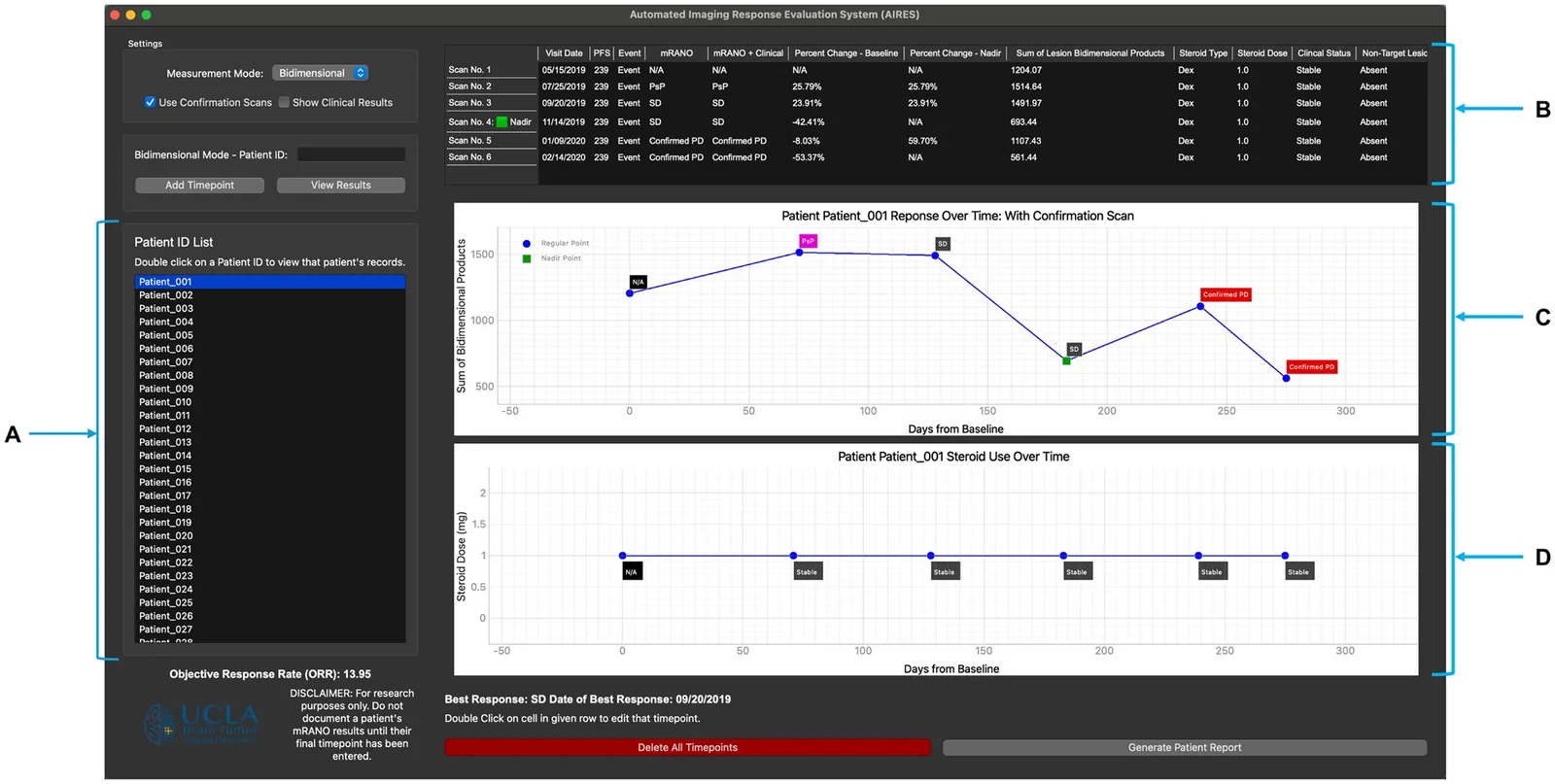
Patient data view. Screenshots of the AIRES and 3D Slicer interfaces illustrating the process of opening 3D Slicer from the AIRES timepoint input form.

Once data have been added to the application, AIRES automatically calculates the response category at each timepoint (progressive disease [PD], pseudoprogression [PsP], stable disease [SD], partial response [PR], complete response [CR]), for both radiographic-only assessments and for combined clinical assessments that incorporate steroid use and neurological status. The application also determines key overall RANO metrics such as progression-free survival (PFS), best response, and the date of best response for each patient. Additionally, the objective response rate (ORR) of the cohort is calculated across all subjects in the database. A list of all patients in the database is displayed (Figure 3A). Double-clicking a patient ID opens that patient’s results, which are presented in a detailed table that includes information for each timepoint (Figure 3B), along with graphs to visualize the change in tumor volume (Figure 3C) and the change in steroid dose over time (Figure 3D).

### AIRES Outputs

**Figure 4.**
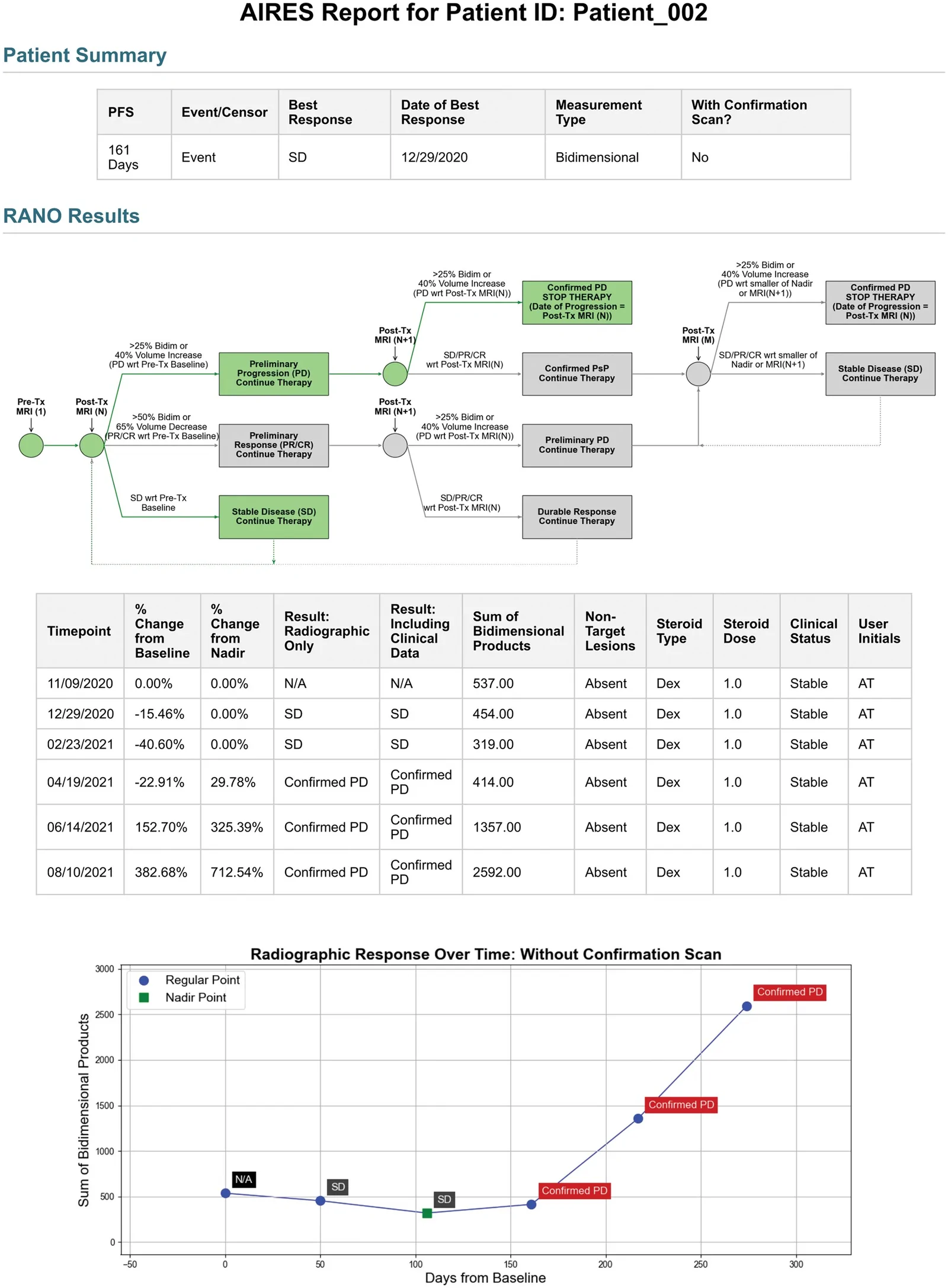
Patient report PDF. Example of an automatically generated patient report, including a summary table, RANO classification flowchart with the algorithm path highlighted, timepoint data table, and a tumor size graph.

Data can be exported from AIRES for offline analysis, which contains all entered timepoints along with the corresponding calculated results. Additionally, a PDF report can be generated for each patient, providing a comprehensive summary (Figure 4). The report includes a table outlining PFS, best response, date of best response, measurement type, and whether confirmation scans were used in the assessment. It also features a flowchart, based on the modified radiographic response assessment rubric,^6^ with the specific decision path taken by the algorithm clearly highlighted. For each timepoint, the report presents a summary table detailing the percent change from baseline and nadir, the radiographic-only assessment, and the integrated assessment that includes clinical data. If path locations of images in NIfTI format are provided for each timepoint (eg, Datasets/timepoint1/post_contrast_T1.nii.gz), the report will also include a mosaic snapshot of the scan for visualization. The PDF report can be viewed directly within the application or downloaded for external use.

### Provenance and Data Integrity

AIRES maintains an audit table for data provenance critical for both research reproducibility and clinical compliance. This audit table provides a detailed, chronological record of all modifications made to the AIRES database, documenting the user’s initials, the justification for any changes or deletions, and the timestamp of the event. This comprehensive logging guarantees the traceability and trustworthiness of all data within the system.

### Installation and Software Availability

While internally tested on exploratory cases and in the NUTMEG subcohort (see “Validation” paragraph below), AIRES should still be considered a prototype system, that requires broader validation on larger cohorts before it can be openly accessible and deployed at scale. As a first step, access to AIRES can be provided upon request through the form at https://btil.dgsom.ucla.edu/resources. AIRES is distributed as a self-contained application and does not require separate installation of Python or other dependencies. It can be easily deployed on both Windows and macOS systems. Installation involves downloading the AIRES package, extracting it to the desired location, and running the application file. The packaged executable distribution allows for simple installation and implements versioning to ensure consistency across institutions. User feedback through our website is welcomed to support ongoing development of the tool.

## Software Validation with Clinical Trial Data

PFS calculations using the mRANO criteria were performed using tumor measurements from 367 scans across 41 patients from NUTMEG, a randomized phase II multicenter clinical trial part of the Cooperative Trials Group for Neuro-Oncology (COGNO)-led (https://cogno.org/) clinical trial. In NUTMEG, elderly patients with newly diagnosed glioblastoma were randomized to nivolumab and temozolomide vs. temozolomide.^15^ For this validation, PFS was calculated based on imaging changes only, without evaluating clinical progression or corticosteroid doses.

Four readers with varying levels of experience (a medical student [MS4], a radiologist, a neurologist, and a data manager [DM]) applied the mRANO criteria to determine PFS (PFS duration and censoring) and presence of objective radiographic response (OR, at any timepoint). Readers were not provided formal training beyond instructions to review the mRANO criteria. A fifth reader, a neuroradiologist with 9 years of experience in neuroimaging and extensive experience in RANO reads of clinical trial data, served as a reference expert reader (ie, ground truth). Additional details regarding reader experience are provided in Supplementary Table S2. Finally, a lab member with background in computer science used AIRES to input measurements and retrieve AIRES-determined PFS and OR. Tumor measurements had been performed externally, prior to this validation test, and were provided to all readers, who therefore applied mRANO criteria on the exact same tumor measurements. All human readers performed fully manual reads using a spreadsheet of patient data, and they were instructed to calculate percentage changes over time and/or graph the data as they considered fit. AIRES-assisted reads were performed by inputting timepoint-specific measurements in the application and retrieving the output PFS and objective response. All readers were blinded to patients’ clinical outcomes and to each other’s reads.

Across the four readers, the fully correct determination of both PFS and objective response was achieved in the following percentage of patients: MS4: 48.78%, Radiologist: 70.73%, Neurologist: 51.22%, DM: 75.61%, compared to the reference expert reader (Table 1). The sensitivity/specificity for the identification of an objective response, compared to the expert reader, were as follows, MS4: 83.33%/97.14%, Radiologist: 100.00%/97.14%, Neurologist: 100.00%/80.00%, DM: 100.00%/97.14%. A correct determination of PFS was achieved in the following percentage of patients: MS4: 53.66%, Radiologist: 73.17%, Neurologist: 60.98%, DM: 75.61%, compared to the reference expert reader. AIRES showed perfect agreement (100%) with the expert reads, both for PFS and OR. Two patients were assessed incorrectly by all four readers, and seven patients were assessed incorrectly by three readers. A qualitative review of the readers’ most common mistakes in these complex cases revealed that errors were often linked to an incorrect interpretation of the confirmation of progression, including incorrect backdating or incorrect censoring in case of regrowth after pseudoprogression without further confirmation. Some other instances had an erroneous handling of non-measurable lesions, including some cases for which non-measurable lesions were erroneously included in the calculation of the tumor burden.

**Table 1.** Performance metrics for objective response classification and progression-free survival across all readers.

Reader	% fully correct reads	Objective response classification		PFS		
		Sensitivity	Specificity	% correct PFS	PFS 12	Median PFS (days)
MS4	48.78	83.33%	97.14%	53.66	64.910%	1229
Radiologist	70.73	100.00%	97.14%	73.17	65.804%	1377
Neurologist	51.22	100.00%	80.00%	60.98	30.684%	186
DM	75.61	100.00%	97.14%	75.61	61.962%	1179
Expert	Reference^*^	Reference^*^	Reference^*^	Reference^*^	52.141%	482
AIRES	100.00	100.00%	100.00%	100.00	52.141%	482

* The PFS and OR determination from the expert reader were used as the reference ground truth to evaluate all other human readers and AIRES.

**Figure 5.**
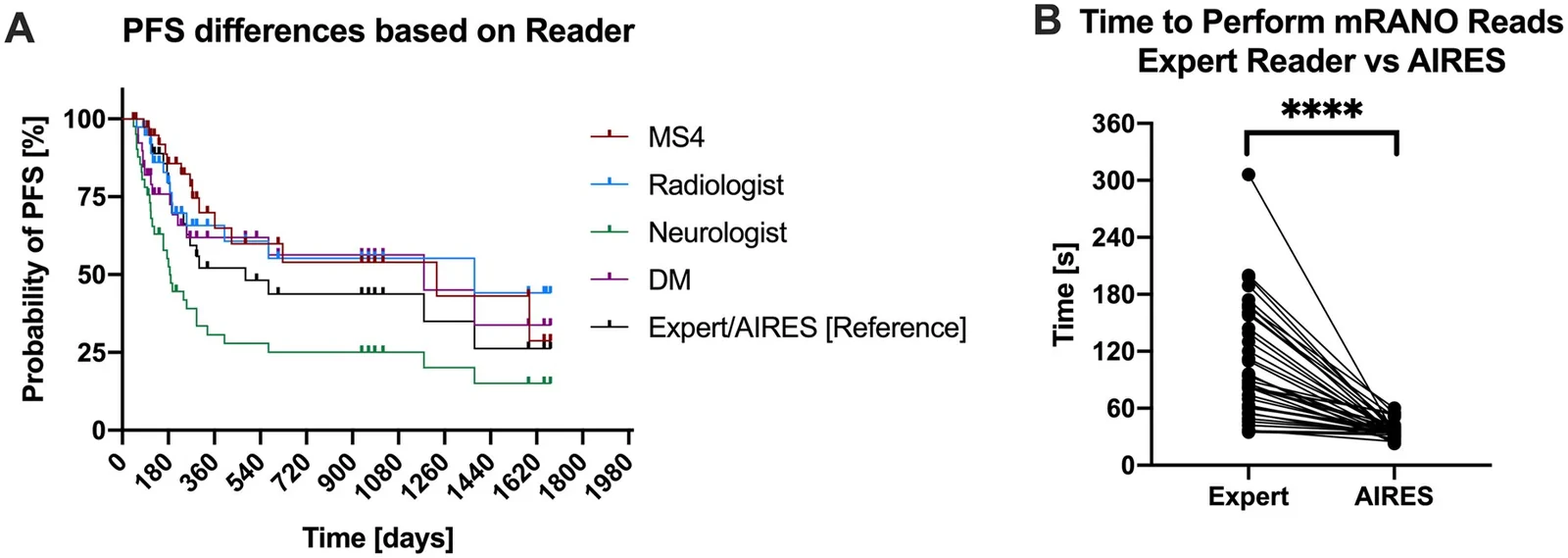
Comparison of manual and AIRES-assisted RANO reads. (A) A Kaplan-Meier curve of progression-free survival in the validation cohort, based on the different readers’ assessments. (B) AIRES-assisted reads were performed significantly faster than fully manual reads performed by an expert reader (37.1 ± 8.6 vs. 107.7 ± 58.6 s; *P *< .0001).

Kaplan Meier curves were created to visualize differences in PFS based on each reader’s assessment (Figure 5A). PFS12 and median PFS were extracted from these curves. PFS12 values (probability of PFS at 12 months) were as follows: Expert/AIRES: 52.141%, MS4: 64.910%, Radiologist: 65.804%, Neurologist: 30.684%, and DM: 61.962%. Median PFS duration were Expert/AIRES: 482 days, MS4: 1229 days, Radiologist: 1377 days, Neurologist: 186 days, DM: 1179 days (Table 1).

Overall, this variability in RANO reads correctness and in the estimated PFS curves suggests that the application of mRANO criteria in non-expert readers may lead to imprecisions when determining imaging endpoints and further justifies the need for an automated tool such as AIRES.

As an additional analysis, a non-parametric paired Wilcoxon test was performed to compare the time to complete the reads between the reference reader’s fully manual reads and the AIRES-assisted reads. The AIRES-assisted workflow was significantly faster (37.1 ± 8.6 vs. 107.7 ± 58.6 s; *P *< .0001), saving around one minute per subject (Figure 5B).

This initial validation has several limitations. This analysis was limited to a relatively small subset from single clinical trial, although multicenter. No discrepancies were found between the calculations performed by the reference reader and AIRES. However, given the complexity of the algorithm and the small cohort size, potential discrepancies during validation of a larger cohort may emerge. Broader validation across independent cohorts with varied imaging protocols will be essential to confirm the generalizability and robustness of AIRES across different clinical contexts. Additionally, the non-reference human readers did not undergo formal training for this project beyond review of the response criteria, which may have contributed to incorrect PFS and OR determinations. While in real-world settings a structured training is typically administered, RANO criteria are complex and it may be challenging for a training to cover all potential instances and scenarios that are encountered when analyzing clinical trial data. Overall, if further validated, AIRES may represent a tool that even untrained personnel can use to perform RANO reads, and even trained personnel may benefit from AIRES to speed up their reads and reduce human errors.

## Conclusion

The AIRES application offers an accessible solution for implementing standardized response assessments. AIRES streamlines a process that is otherwise time-consuming and error-prone, while maintaining transparency and reproducibility. By automating complex response criteria, AIRES may lower costs by reducing the need for specialized personnel training and manual reads. Beyond its central usefulness in prospective clinical trials, AIRES is a useful tool for a fast and reliable assessment of RANO imaging endpoints in retrospective research^4^ and in the clinical practice, where a cautious and flexible RANO evaluation may be insightful.^10^

AIRES was built primarily for the mRANO criteria, but future development will aim to expand AIRES beyond mRANO to incorporate additional response frameworks such as RANO-BM and RANO 2.0 for non-enhancing and mixed tumors. Future versions may also include additional endpoints such as disease control rate (DCR), individual tumor growth rates, and more in-depth visualization. Deployment of AIRES within clinical trial sites, such as GBM AGILE, is planned. This collaborative approach facilitates feedback from end-users to guide ongoing development. Broader deployment would also improve data integrity and consistency across sites while further reducing costs through standardized, reproducible radiological reads.

Overall, this study demonstrates that automation of radiographic response assessment using AIRES is both feasible and beneficial. Widespread adoption of such tools could enhance consistency across clinical sites, reduce operational costs, and support more reliable and efficient evaluation of treatment efficacy in glioblastoma and other central nervous system tumors.

## Supplementary Material

npag039_Supplementary_Data

## Data Availability

Patient data from the NUTMEG trial, which were used for software validation, can be made available upon request, pending approval from the sponsor and under data sharing agreements.

## Application Availability

The AIRES application can be provided upon request through the dedicated form at https://btil.dgsom.ucla.edu/resources.
