# Supplementary material for: Automated imaging response evaluation system: Prototype software tool for standardized radiographic response assessment in glioblastoma clinical trials
Source: Neurooncol Pract. 2026 May 14;13(5):867–76. doi: 10.1093/nop/npag039 (PMC13578499; doi:10.1093/nop/npag039)
Supplement: Supplementary file 1 [file npag039_supplementary_data.pdf]

# Automated Imaging Response Evaluation System (AIRES): prototype software tool for standardized radiographic response assessment in glioblastoma clinical trials

*(Supplement prepared for Neuro-Oncology Practice)*

| patient_ID | visit_date | lesion_1_diam_1 | lesion_1_diam_2 | … | lesion_5_diam_1 | lesion_5_diam_2 | volume | steroid_type | steroid_dose | clinical_status | non_target_lesions |
|---|---|---|---|---|---|---|---|---|---|---|---|
| 001 | 2018-04-20 | 71.94 | 47.26 | | N/A | N/A | 42682.7 | Dex | 4 | Stable | Absent |
| 001 | 2018-06-13 | 69.77 | 47.17 | | N/A | N/A | 41445.4 | Dex | 4 | Stable | Absent |
| 002 | 2019-02-27 | 46.1 | 28.18 | | N/A | N/A | 18918.09 | Dex | 4 | Stable | Absent |
| 002 | 2019-04-04 | 68.36 | 25.6 | | 13.1 | 10.2 | 47232.91 | Dex | 8 | Decline | Absent |
| 003 | 2019-01-02 | N/A | N/A | | N/A | N/A | 2004.94 | N/A | 0 | N/A | Absent |
| 003 | 2019-03-07 | 34.93 | 22 | | N/A | N/A | N/A | Dex | 24 | N/A | Absent |

**Supplementary Table 1. Example CSV Format for Upload.** The table shows the required column structure for importing data into AIRES from CSV files. Column order and formatting must match exactly for successful upload. Example entries illustrate acceptable values for each column.

| Reader | How much knowledge of mRANO did you have before this project? | Have you ever performed mRANO reads for research projects or clinical trials before? | How long did you study the mRANO manuscript immediately before starting the reads? |
|---|---|---|---|
| Medical Student (MS4) | None | Never | 1-2 Hours |
| Radiologist | Some | Never | 0.5-1 Hour |
| Neurologist | Moderate | Yes. >10 and <50 patients | 0.5-1 Hour |
| Data Manager (DM) | Some | Never | 1-2 Hours |
| Expert Reader | Extensive | >100 patients | 0 Hours |

**Supplementary Table 2. Reader Experience with mRANO.** The table shows information about each reader's knowledge of the mRANO criteria prior to this project. The values were obtained from a questionnaire provided to each reader after the completion of their reads.
